# Siderophores: A Case Study in Translational Chemical Biology

**DOI:** 10.1021/acs.biochem.4c00276

**Published:** 2024-07-23

**Authors:** Andrew R. LeBlanc, William M. Wuest

**Affiliations:** Department of Chemistry, Emory University, Atlanta, Georgia 30322, United States

**Keywords:** siderophores, secondary metabolites, natural products, antibiotics

## Abstract

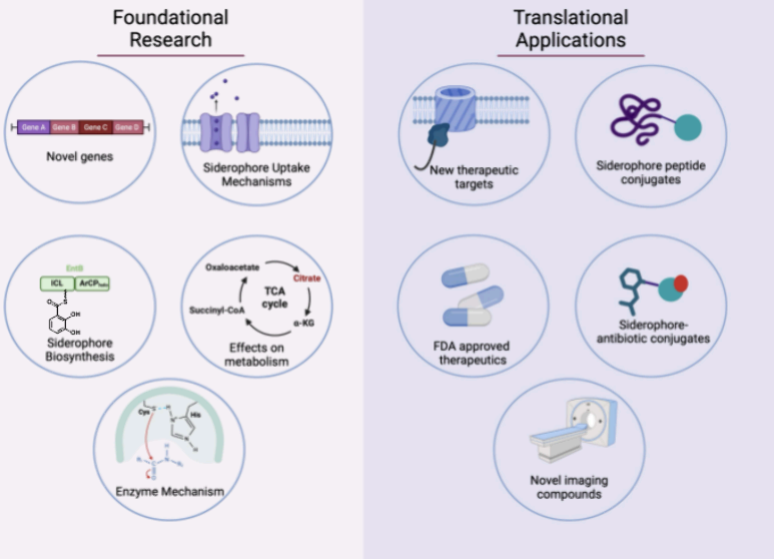

Siderophores are metal-binding secondary metabolites that assist in iron homeostasis and have been of interest to the scientific community for the last half century. Foundational siderophore research has enabled several translational applications including siderophore-antibiotic and siderophore-peptide conjugates, identification of new antimicrobial targets, advances in disease imaging, and novel therapeutics. This review aims to connect the basic science research (biosynthesis, cellular uptake, gene regulation, and effects on homeostasis) of well-known siderophores with the successive translational application that results. Intertwined throughout are connections to the career of Christopher T. Walsh, his impact on the field of chemical biology, and the legacy of his trainees who continue to innovate.

## Introduction

Molecules within living systems can be broken up into two classes: primary metabolites and secondary metabolites. Primary metabolites are molecules necessary for growth, reproduction, or development. These molecules include amino acids, nucleosides, enzymes, vitamins, and carbohydrates and are typically the central focus of traditional biochemistry. In contrast, secondary metabolites, often termed natural products, are small molecules that facilitate beneficial interactions between a living system and its surroundings. Over time, evolutionary pressures have selected for secondary metabolites with a specific biological function. In bacteria, natural products have been shown to possess antibacterial and antifungal activity,^1^ inhibit biofilm formation,^2^ and regulate reactive oxygen species.^3^ The career of Christopher T. Walsh was epitomized by his use of chemistry to understand biological systems and, in particular, the biosynthesis and regulation of secondary metabolites. This approach of leveraging chemistry to better understand biology was termed “chemical biology”. Although the definition of chemical biology is ever changing, Walsh put it best.

*“Think chemically, act biologically”*.

–Christopher Walsh^200^

Through a chemical biology lens, Walsh spent his career studying a vast array of topics including suicide inhibitors,^4^ mechanisms of antimicrobial resistance,^5^ siderophores,^6^ and the enzymology of natural product biosynthesis.^7^ Discovering, exploring, and understanding these biological molecules and processes lends itself naturally to translational research. It was within this arena that Walsh was a pioneer, showing how basic discoveries can lead to novel therapeutics. Throughout his career, he was involved in multiple biotech companies including ImminoGen Inc., LeukoSite, Vicuron Pharmaceuticals, TransForm Pharmaceuticals, Inc., and Kosan Biosciences, Inc., all of which were successful, and many were acquired by larger companies (i.e., Pfizer, Johnson & Johnson, and Bristol-Myers Squibb).^8^ The desire to conduct translational research was also a driving force in his independent academic career. In 1987, Walsh was recruited away from the biology department at MIT to a position at the Harvard Medical School. In his 2010 memoir,^8^ Walsh states that one factor for leaving MIT was the “pull to learn more about therapeutics and human biology by going to/joining a medical school.” Furthermore, Chris imparted the importance of translational research onto the more than 250 graduate students and postdocs he mentored throughout his career. Many of his former trainees have proceeded to embody his approach to translational research with multiple going on to start their own successful biotech companies. Specific examples include Peter Schultz (Walsh Lab postdoctoral scholar, 1984–1985) founder of Affymax Research Institute, Symyx Therapeutics, and Wildcat Technologies and Greg Verdine (Walsh Lab postdoctoral scholar, 1986–1988) who launched FogPharma, LifeMine, Warp Drive Bio, and Wave Life Sciences among others.

**Figure 1:**
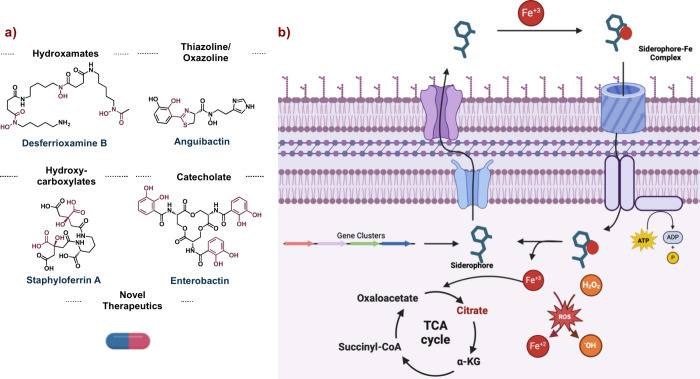
Siderophore overview. a) Highlighting the different classes of siderophores and b) their cellular intake, biosynthesis, and effect on different cellular processes. Abbr. TCA, tricarboxylic acid; ROS, reactive oxygen species.

Throughout his career Walsh pioneered the biosynthesis of siderophore natural products. During my time in the lab (W.M.W.), spanning from 2008 to 2011, several projects were underway investigating the biosynthetic machinery that constructed these essential molecules of life. Siderophores are secondary metabolites that bind metals with high affinity and play an essential role in bacteria by aiding in the regulation and control of iron uptake. Iron is essential for all life forms and is involved in multiple bacterial processes such as the citric acid cycle,^9^ electron transport chain,^10^ biofilm formation,^11^ regulation of reactive oxygen species,^3^ and small molecule biosynthesis. The amount of iron required for living systems is around 10^–7^ to 10^–5^ M.^12^ However, at physiological pH (7.4), iron hydrate Fe(OH)_3_ is highly insoluble at concentrations as low as 10^–18^ M.^13^ Bacteria have evolved to synthesize siderophores to regulate and control the concentration of intercellular iron and as a result regulate different cellular processes. There are four main structural classes of siderophores (Figure 1a), each with unique biological properties: hydroxamates, thiazoline/oxazolines, hydroxycarboxylates, and catecholates. All classes of siderophores chelate the metal through two proximal heteroatoms, and in doing so, increase the water solubility of iron. The siderophore-iron complex can then pass into the cell before being used for a wide variety of processes (Figure 1b), Since their discovery, siderophores have been of interest to the biochemical community resulting in over 17,000 publications (SciFinder^©^, May 2024) and are a gateway to understanding chemical transformations within living organisms. Examining siderophore function in biological systems has been instrumental across multiple fields including the discovery of different regulatory pathways. These foundational discoveries have led to translational applications in medicine, imaging, and probe development.

“*I have been always fascinated with biology and its intersection on medicine, but I think morning, noon, and night, as a chemist. I am really about the molecules of life. . .”*

–Christopher Walsh^14^

This review will highlight the foundational and subsequent translational research of siderophore biosynthesis, much of which was pioneered by Walsh and the multiple trainees he mentored over the years. Specifically, this review will walk through the foundational research of four well-studied and chemically distinct siderophores (desferrioxamine B, acinetobactin, staphyloferrin A-B and enterobactin), and their applications to translational research via novel therapeutics, and throughout, acknowledge and highlight the work of both Walsh and his trainees.

## Hydroxamates: Desferrioxamine B

**Figure 2:**
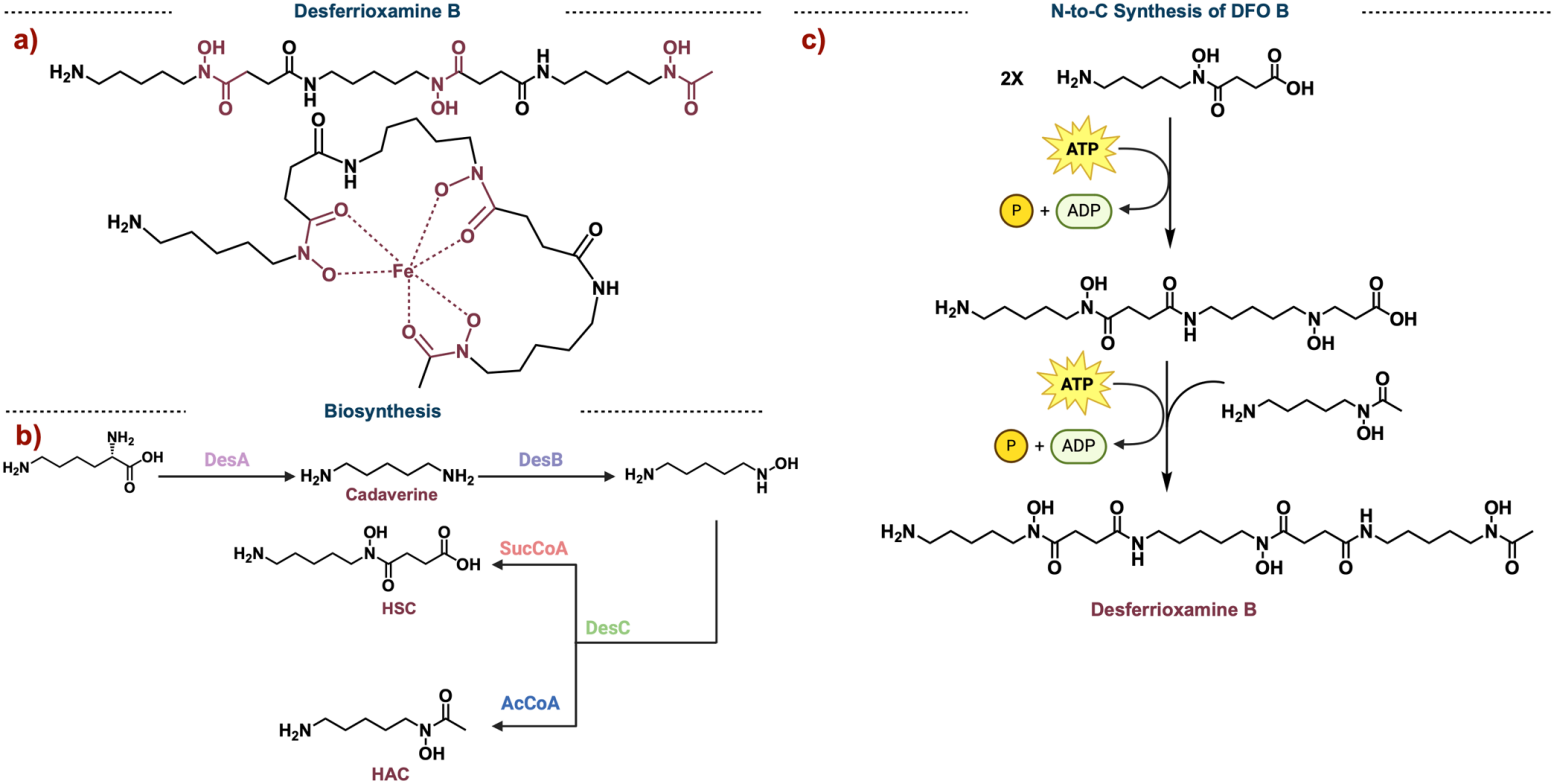
a) Structure of desferrioxamine B (DFO B) and its binding mode with iron. b) The biosynthesis of DFO B precursors. c) Synthesis of DFO B originating at the N-terminus.

Desferrioxamine B (DFO B), a hydroxamate (*N*-hydroxyl amide) siderophore, was first isolated in 1958 from *Streptomyces pilosus* and shown to have medicinal applications (Figure 2a).^15^ In 1968, DFO B was approved by the United States Food and Drug Administration (FDA) for the treatment of iron poisoning and hemochromatosis.^16^ Unsurprisingly, DFO B binds iron in the body, solubilizing it to be excreted. In 2019, DFO B was placed on the World Health Organization (WHO) list of essential medicines.^17^ The foundational understanding of DFO B, along with other siderophores, often begins by interrogating their biosynthetic machinery, an approach that Walsh pioneered.

The biosynthetic precursors of DFO B are lysine, acetyl-CoA, and succinyl-CoA. *L*-Lysine undergoes a decarboxylation by DesA to give rise to cadaverine. DesB then selectively oxidizes one of the primary amines to the *N*-hydroxyl amine.^18^ Next, DesC along with acetyl-CoA or succinyl-CoA gives rise to the extended *N*-hydroxy-*N*-acetyl-cadaverine (HAC) or *N*-hydroxy-*N*-succinyl-cadaverine (HSC) (Figure 2b). Finally, DesD performs an ATP-dependent condensation with molecules HAC and HSC. In the case of DFO B, two molecules of HSC and one molecule of HAC are joined.^19^ Work by Wencewicz (Walsh Lab postdoctoral scholar, 2011–2013) and co-workers set out to understand if DesB synthesized DFO B from the N-to-C or C-to-N terminus. Using synthetically prepared dimers and isotope studies, they found that DFO B is constructed from the N-to-C terminus. That is, HSC is activated by ATP to form the HSC-AMP adduct which dimerizes to give the HSC dimer. The terminal carboxylic acid of the dimer is then activated, and HAC is added in to furnish DFO.^19^

**Figure 3:**
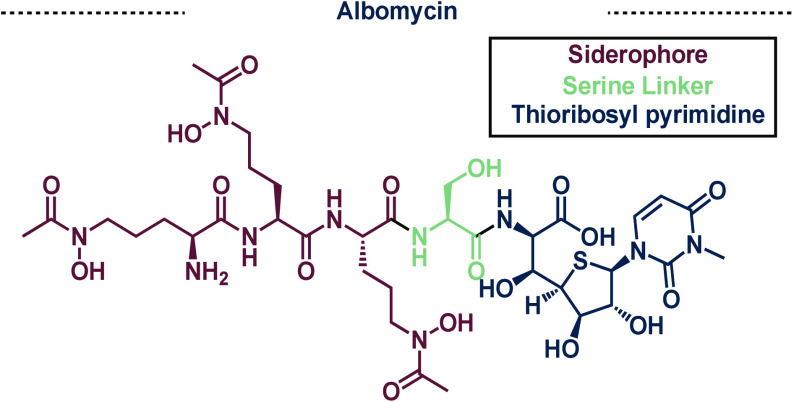
Structure of the naturally occurring albomycin natural product which possesses a siderophore, a cleavable serine linker, and active thioribosyl pyrimidine.

Albomycin is another hydroxamate siderophore natural product that has been extensively studied over the years. Although first isolated in 1947 from *Streptomyces griseus*, the complete chemical structure was unknown until 1982.^20,21^ Albomycin is a chimeric natural product that possesses a trihydroxamate motif, serine linker, and thioribosyl pyrimidine structure (Figure 3).^20^ These three distinct chemical features make albomycin the first characterized siderophore antibiotic conjugate (SAC). SACs are antibiotics that are covalently linked to a siderophore motif, tricking the cell into taking up the complex molecule. Once inside, the antibiotic portion of the SAC kills the cell. This overall strategy has been coined “trojan horse antibiotics” as it exploits the native machinery of the bacteria, akin to the trojan horse in Greek mythology. This approach has been specifically highlighted in Gram-negative bacteria due to the challenges in crossing the outer membrane. Albomycin is extremely potent toward *Escherichia coli* with an MIC of 5 ng/mL highlighting the efficacy of such an approach.^22^ Specifically, albomycin passes through both the inner and outer membranes before it is cleaved at the serine linker by peptidase to produce the free thioribosyl pyrimidine, which was later shown to be a tRNA synthetase inhibitor.^23−25^

**Figure 4:**
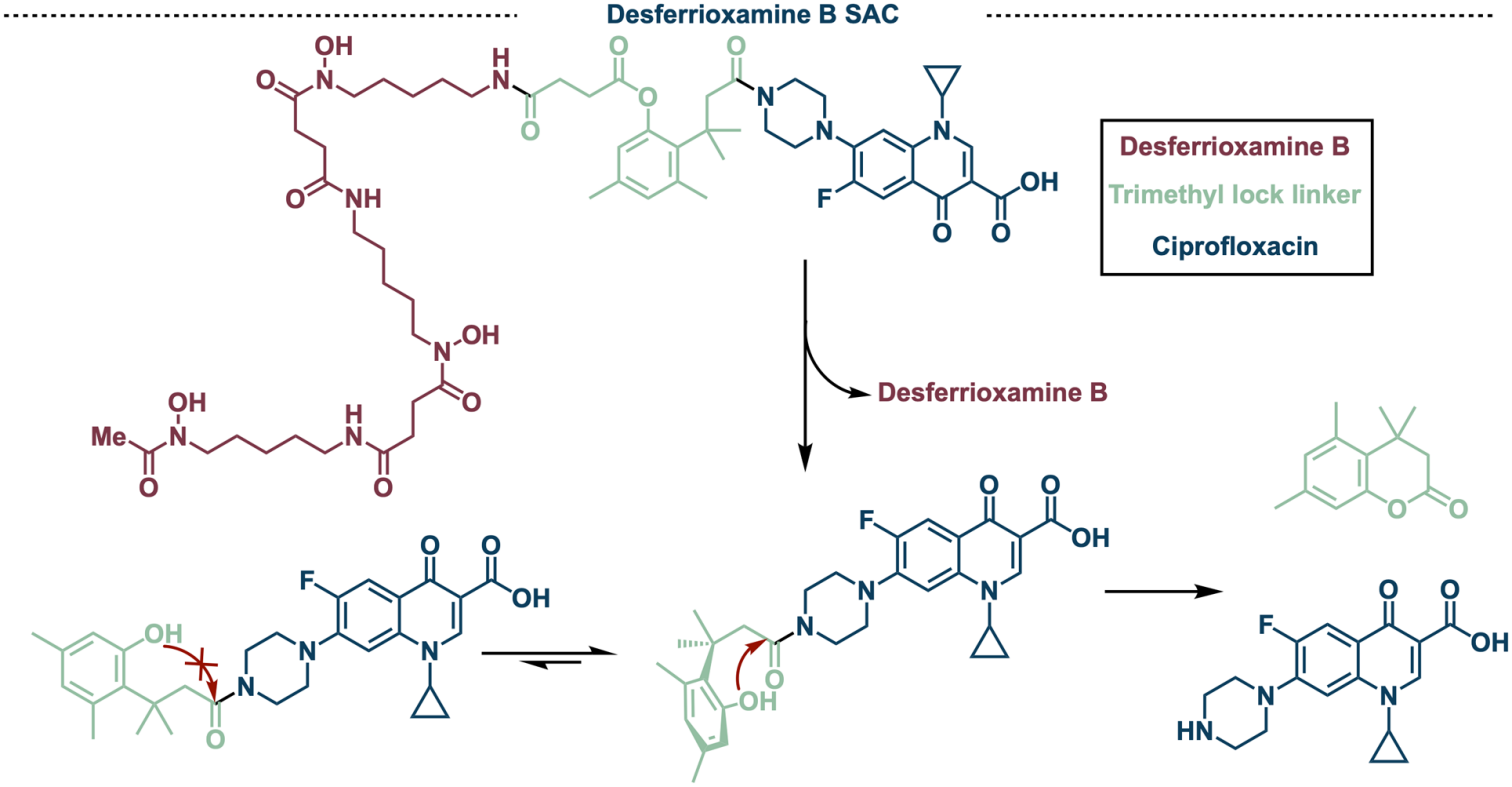
Strategically designed desferrioxamine B-ciprofloxacin SACs with a trimethyl lock linker to increase the rate of release.

Due to the naturally occurring hydroxamate siderophore antibiotic conjugates, along with the clinical success of desferrioxamine B, synthetic SACs have been at the forefront of translational SAC discovery (Figure 4). To date, desferrioxamine B has been coupled with many different antibiotics such as fluoroquinolones, sulfonamides, and ß-lactams.^26^ Specifically, DFO B conjugated to Loracarbef (ß-lactam) showed a remarkable increase in potency against *Micrococcus luteus* with a potency of 1 nM compared to 0.39 μM of Loracarbef alone.^26^ One DFO-ciprofloxacin conjugate that stands out was developed by the Miller lab at the University of Notre Dame.^26^ This conjugate uses an esterase/phosphatase dependent drug release mechanism, like that of the naturally occurring albomycin. The strategically designed linkage possessed a masked phenol that could be cleaved *in situ* by an esterase or phosphatase enzyme. The free phenol can then undergo an intramolecular esterification, releasing the active drug. To increase the rate of the cleavage, Ju and Miller leveraged a foundational “trimethyl lock” that was first reported in 1972 by Milstien and Cohen.^27^ The trimethyl lock takes advantage of fundamental physical organic properties, a passion of Walsh’s, to preposition the molecule for a faster intermolecular reaction.^28^ Importantly, this cleavage based approach does not compromise potency for the structural addition of the siderophore. In this case, their cleavage-based SACs exhibited moderate activity but were unable to outperform the parent antibiotic. This decreased antimicrobial activity is likely attributable to the poor enzymatic hydrolysis of these SACs.

## Oxazoline/Thiazoline: Pyochelin and Acinetobactin

**Figure 5:**
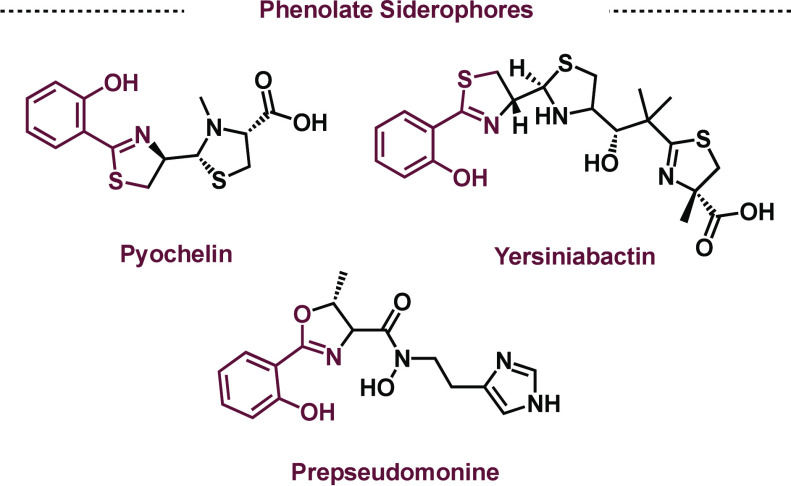
Examples of oxazoline/thiazoline siderophores.

**Figure 6:**
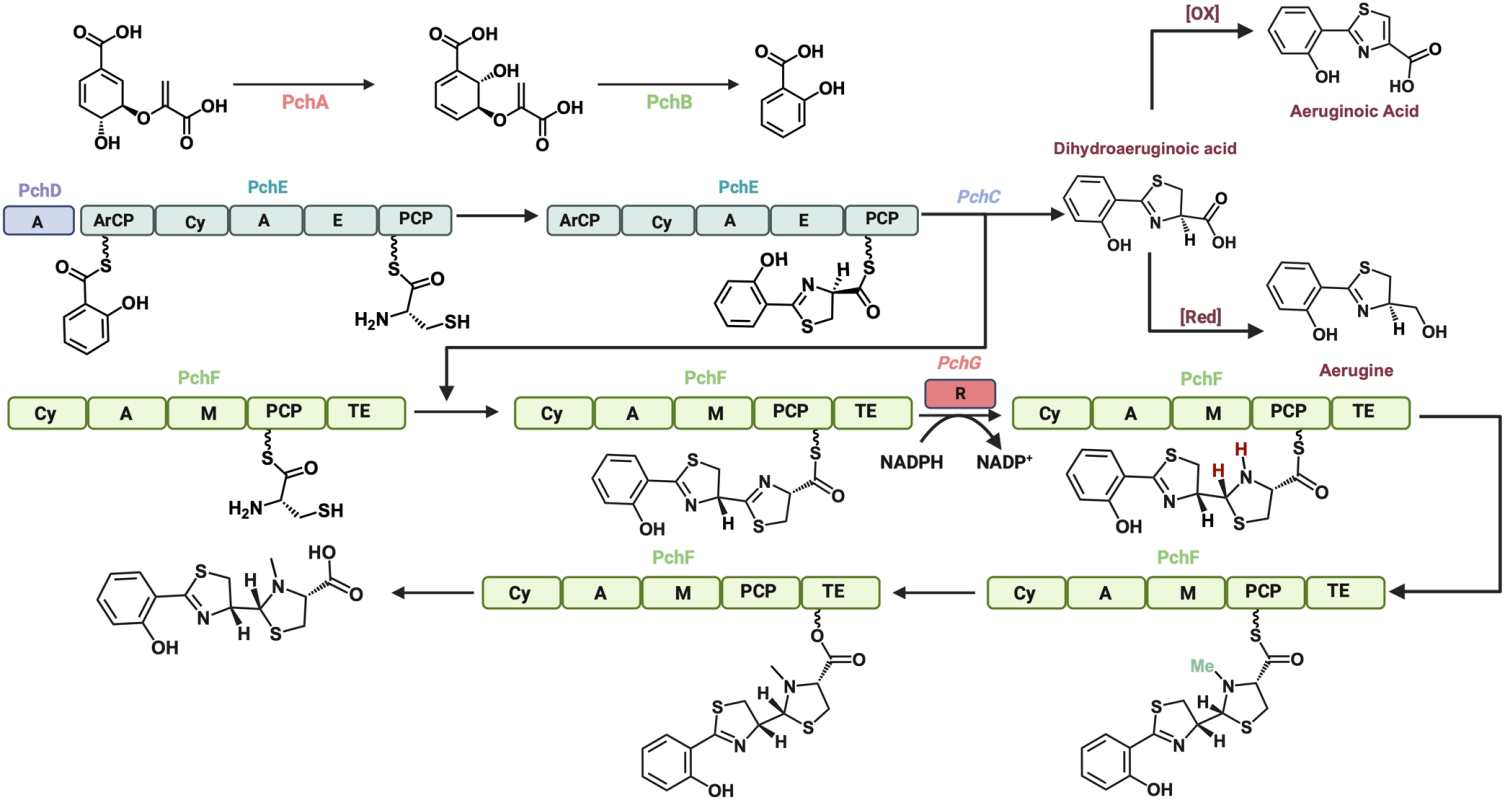
Biosynthesis of pyochelin, highlighting the shunt products isolated.

Nature has realized that by appending an oxazoline/thiazoline motif adjacent to a phenolate it creates a tight metal chelation with the oxygen of the phenol and nitrogen of the oxazoline/thiazoline. One of the most well studied phenolate-thiazoline siderophores is pyochelin, first isolated from *Pseudomonas aeruginosa* in 1981 (Figure 5).^29^ The biosynthesis of pyochelin is a fundamental example of a nonribosomal peptide synthetase (NRPS). NRPSs are large enzymatic assembly lines that construct a wide range of chemically diverse peptides. These NRPS assembly lines can generally be broken up into four functions: 1) initiation, 2) elongation, 3) tailoring, and 4) termination. Initiation is the process of loading the biosynthetic precursors onto a peptide carrier protein (PCP) via adenylation (A). Elongation occurs primarily through the function of condensation (C) domains (Figure 6). Tailoring of these structures can be accomplished through a variety of ways including epimerization (E), methylation (MT), and cyclization (Cy). Finally, termination typically involves a thioesterase domain (TE) which releases the peptide from the enzymatic assembly line although reductive cleavage has also been demonstrated.^30^ Importantly, the peptide can either be cleaved to form a linear product or undergo a cyclization to form the corresponding macrocycle.^7,31^ Pyochelin biosynthesis requires 2-hydroxybenzoate, which is not naturally occurring, but can be prepared from chorismate in two enzymatic steps by PchA and PchB. PchA catalyzes the isomerization of chorismate to isochorismate. PchB then catalyzes an elimination-like reaction to expel pyruvate and furnish 2-hydroxybenzoate. Next, the initiation of the NRPS takes place, where PchD activates 2-hydroxybenzoate and loads it onto the aryl carrier protein (ArCP) of PchE. Additionally, *L*-cysteine is loaded onto the peptide carrier protein (PCP) domain of PchE (Figure 6).^32^ The first elongation step is the amide formation between *L*-cysteine and 2-hydroxybenzoate to form the linear precursor. Then, a dehydrative cyclization and epimerization of the alpha-position to form the necessary thiazoline motif.^33^ On a separate protein, PchF, *L*-cysteine is loaded on its PCP domain. The adjacent cyclase of PchF cyclizes *L*-cysteine from its own PCP domain and the 2-hydroxythiazoline of PchE to form the tricyclic core of pyochelin. A tailoring enzyme, PchG, selectively reduces the newly formed thiazoline.^34^ After reduction, a methylation domain within the PchF protein methylates the secondary amine. Finally, transfer of the PCP-bound pyochelin to the TE domain, and hydrolysis, produces pyochelin.

Over the years many natural products have been identified as shunt products of pyochelin biosynthesis. A major shunt product of pyochelin is the thioester hydrolysis of PchE, catalyzed by PchC, to form dihydroaeruginoic acid (DHA). Interestingly, these shunt products retain the same stereochemistry from the *L*-cysteine suggesting that cleavage takes place prior to thioester hydrolysis. DHA can then undergo redox manipulations to form other natural products including aerugine, aeruginoic acid, aeruginaldehyde, and aeruginol.^35^ Work by Clardy (Christopher T. Walsh Professor of Biological Chemistry and Molecular Pharmacology, Harvard Medical School) and co-workers showed that these pyochelin shunt products along with intermediates in the biosynthesis of pyrrolnitrin react via a nonenzymatic Pictet-Spengler reaction to form a novel class of natural products—pyonitrins.^36^ Later work by Wuest (Walsh Lab postdoctoral scholar, 2008–2011) and co-workers independently synthesized these pyochelin shunt products and found they were able to bind iron with a K_d_ ranging from 16 to 206 μM. Moreover, these chelated iron side products were able to rescue the growth of a *Pseudomonas aeruginosa* double knockout strain unable to produce pyoverdine or pyochelin (ΔpvdDΔpchEF).^37^

“*Nature loves heterocycles, nitrogen heterocycles*.”

– Christopher Walsh^38^

**Figure 7:**
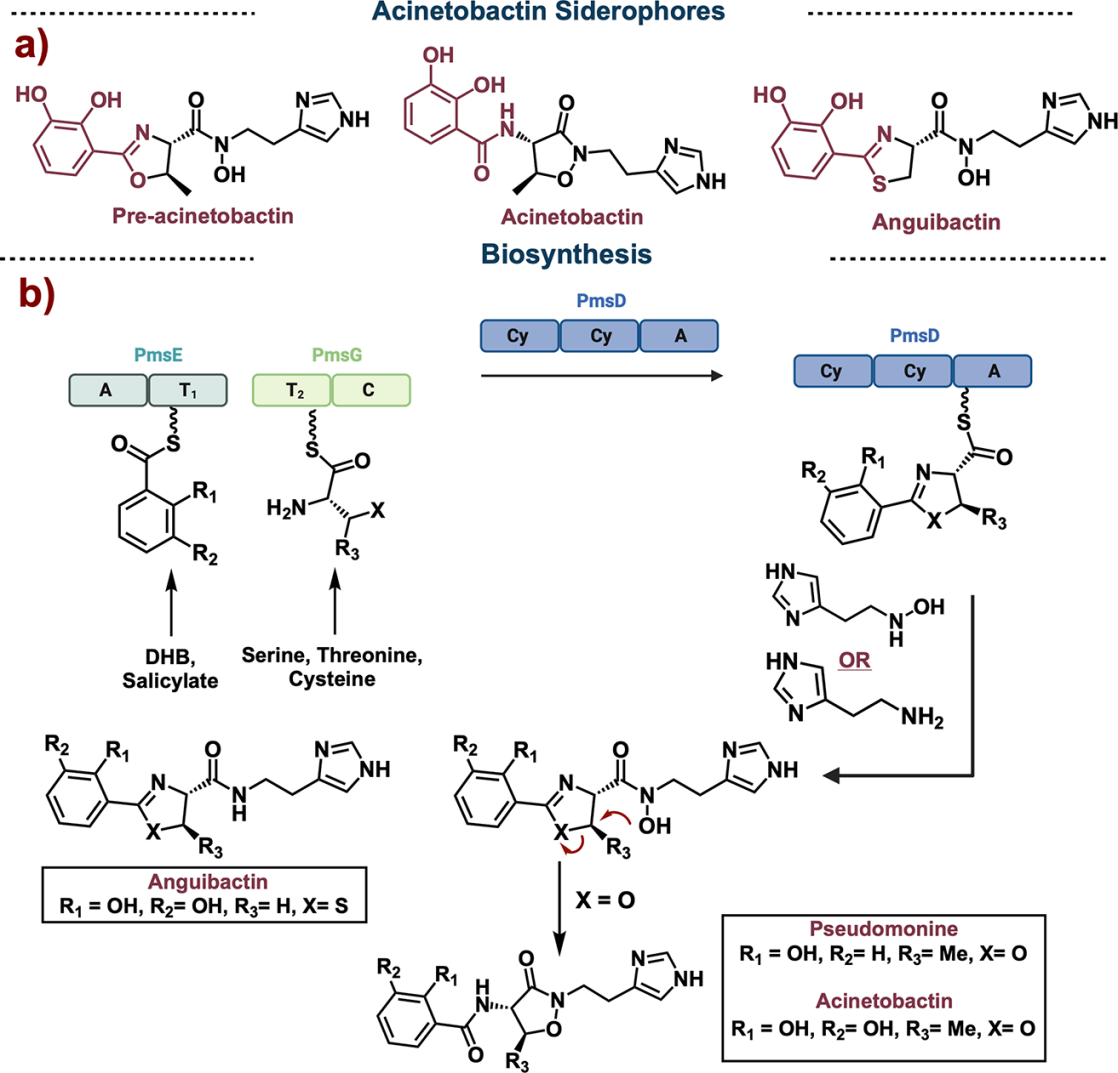
a) Related acinetobactin siderophores. b) Divergent biosynthesis of acinetobactin from the same enzymatic assembly line.

**Figure 8:**
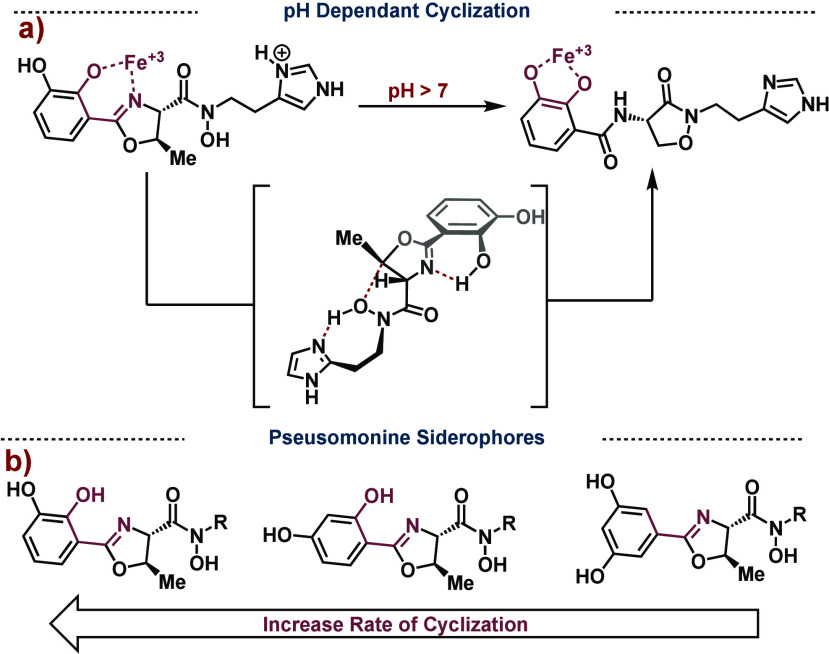
a) The pH dependent cyclization of Pseusomonine described by Wencewicz. b) Rate of cyclization based on electronics.

Oxazoline/thiazoline siderophores have also been identified as interesting biosynthetic intermediates in the biosynthesis of pseudomonine and acinetobactin. For the biosynthesis of pseudomonine, 2-hydroxybenzoate is loaded onto the T domain of PmsE and threonine is loaded onto PmsG. Then, a cyclization event by PmsD and final amide bond formation with *N*-hydroxy histamine gives rise to prepseudomonine.^39^ Work by Walsh and Sattely (postdoctoral scholar, 2007–2010) showed that prepseudomonine rapidly undergoes a nonenzymatic cyclization between the oxazoline and adjacent hydroxylate to produce pseudomonine.^40^ Wuest, Sattely, and Walsh also showed that the enzymatic machinery in the biosynthesis of pseudomonine could accommodate serine and cysteine on the PmsG domain giving rise to unsubstituted oxazoline and thiazoline. Moreover, DHB can be loaded onto PmsE in place of salicylate. Finally, PmsG recognizes histamine in the final amidation (Figure 7b).^41^ Importantly, the lack of the hydroxamate prevented the later cyclization giving rise to novel siderophore compounds. It was also found that the thiazoline motif (anguibactin) did not undergo cyclization. These findings were later synthetically and computationally validated. Overall, the promiscuity of PmsD-G allowed one enzymatic assembly line to access three siderophores and multiple other unique metabolites.^41^ The unique nonenzymatic cyclization in the biosynthesis of pseudomonine and acinetobactin has since been studied in much detail. Kim and Wencewicz showed that both acinetobactin and preacinetobactin promotes the growth of *Acinetobacter baumannii* and are capable of binding Fe^III^ in a 2:1 ratio. Both Kim and Wencewicz independently synthesized a series of acinetobactin analogs to uncover the structure–activity relationship of acinetobactin.^42,43^ They found that preacinetobactin only requires the 2-hydroxyl and adjacent oxazoline to bind iron. Acinetobactin itself, however, lacks the oxazoline motif and therefore requires the 2,3-diol (catechol) to chelate iron. Wencewicz demonstrated that preacinetobactin has a prolonged half-life at low pH (pH < 6.5). At pH > 7, however, preacinetobactin rapidly cyclizes which is due to the adjacent imidazole motif acting as a pH regulator (Figure 8a).^44^ Under slightly basic conditions, the imidazole is deprotonated (p*K*_a_ = ∼6.8) and acts as a hydrogen bond acceptor to the adjacent N-hydroxamate which increases the nucleophilicity and causes rapid cyclization. Under acidic conditions, the imidazole is in its protonated form and unable to act as a hydrogen bond acceptor, slowing the rate of cyclization. Wencewicz also showed electronics of the phenyl ring can dictate the rate of cyclization. Specifically, they found dihydroxylation at the 2,3-positions of the phenyl ring, as shown in acinetobactin, gives the highest rates of cyclization compared to the 2,4- and 3,5-diols, respectively (Figure 8b).^43^

## α-Hydroxycarboxylate Siderophores: Staphyloferrin

**Figure 9:**
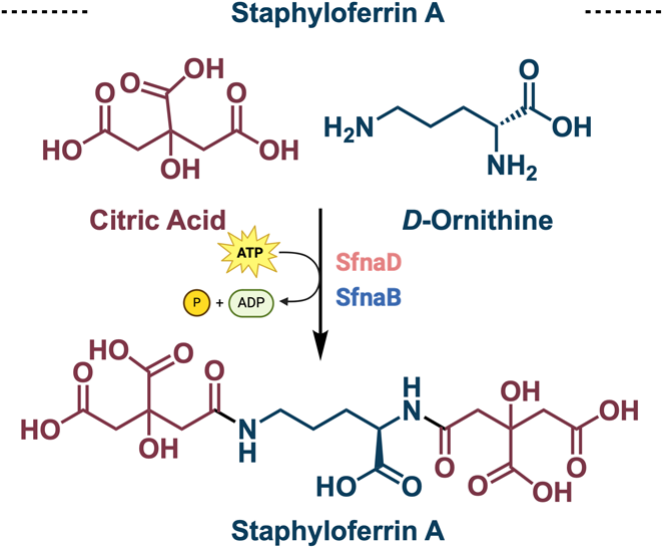
Biosynthesis of staphyloferrin A and related gene cluster.

**Figure 10:**
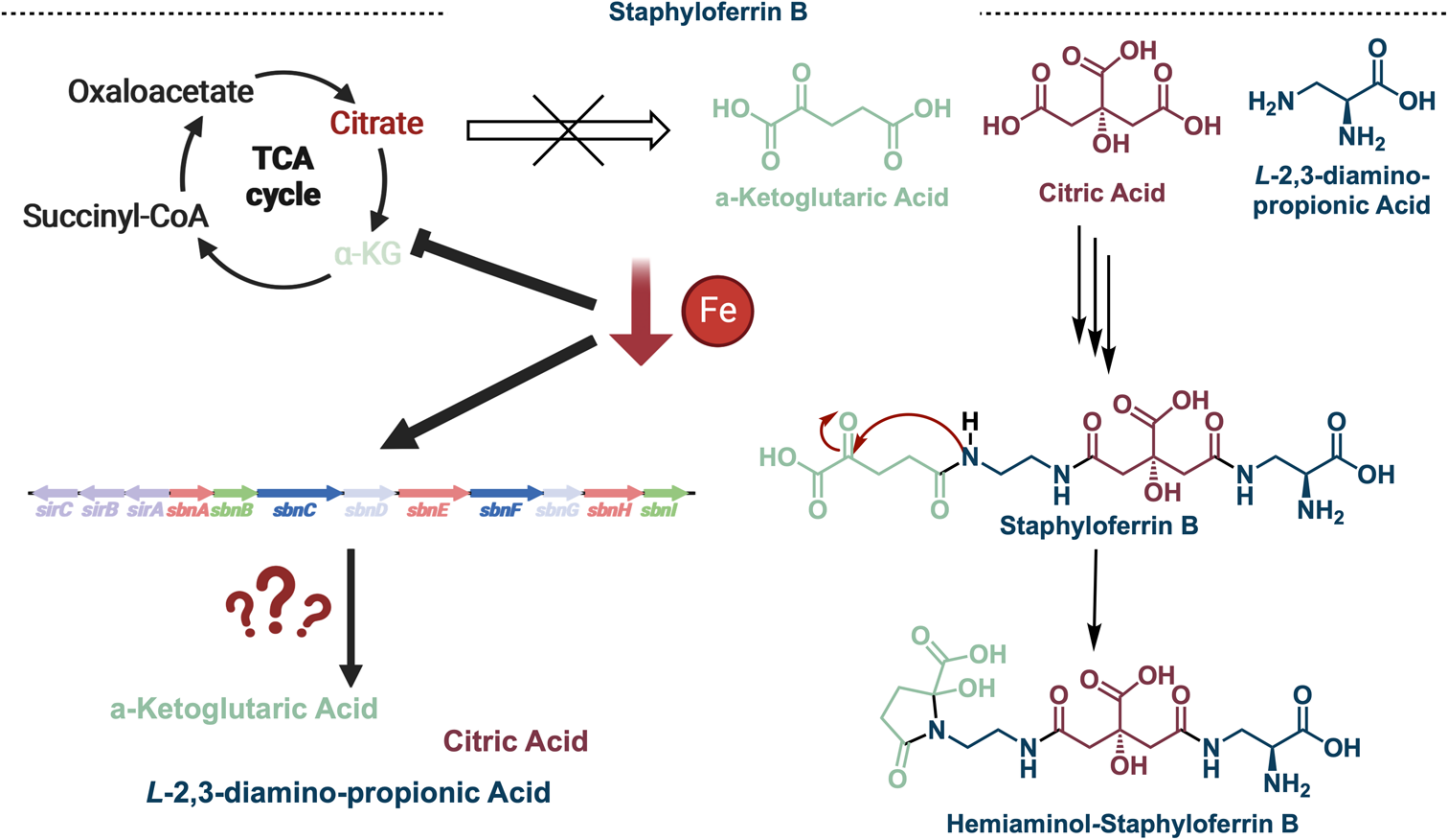
Complex relationship between the building blocks of staphyloferrin B, iron concentration, and the TCA cycle suggesting additional enzymatic machinery is necessary to synthesis α-KG, *L*-DAP, and citric acid under limited iron conditions.

α-Hydroxycarboxylate siderophores are made through NRPS machinery and chelate metals through the carbonyl and adjacent α-hydroxy motif. Possibly the most well studied α-hydroxycarboxylate are staphyloferrins A and B, which were isolated from *Staphylococcus hyicus* in 1990 and 1994, respectively.^45,46^ Staphyloferrin A possesses two citric acid fragments and one connecting ornithine motif. In 2009, Balibar (Walsh Lab graduate student, 2003–2007) and co-workers further probed the biosynthesis of staphyloferrin A by isolating its gene cluster.^47^ Expectedly, two synthetase enzymes, SfnaB and SfnaD, catalyze the addition of citric acid onto d-ornithine in an ATP dependent manner (Figure 9).^47^ Compared to staphyloferrin A, staphyloferrin B possesses an intrinsically more complex chemical structure. A later isolation of staphyloferrin B from *Ralstonia eutropha* in 1999 also isolated it as a cyclic isomer.^48^ This increased chemical complexity have made the biosynthesis of staphyloferrin B (SB) of great interest. SB can be traced back to three distinct building blocks whose biosynthesis has been studied in detail: *L*-2,3-diaminopropionic acid (*L*-DAP), α-ketoglutaric acid (α-KG), and citric acid. All three of these SB building blocks are found within the citric acid (TCA) cycle. However, iron starvation in bacteria redirects its central metabolism which limits the production of these necessary metabolites.^49,50^ The lack of these building blocks when siderophore production is necessary suggested that there may be another pathway to access *L*-DAP, α-KG, and citric acid (Figure 10).^51^

Foundational work from Dale and co-workers identified the gene cluster, *sbnA-I* responsible for its biosynthesis, which lead to others studying it.^52^ Through examining genetics, enzyme kinetics, X-ray crystallography, and traditional biochemistry experiments, Beasley and Kobylarz showed that the genes sbnA and *sbnB* encoded for the biosynthesis of *L*-DAP and α-KG under iron starvation for the synthesis of SB.^53−55^ These initial findings, however, did not account for the synthesis of citric acid under iron starvation. Work by Heinrichs in 2012, found that the enzyme SbnG catalyzes a metal independent aldol reaction between acetyl-CoA and oxaloacetate to produce citric acid.^56^ Homology studies found a similar iron independent aldolase enzyme in other gene clusters for similar citric acid containing siderophores.^56^ Surprisingly, the biosynthetic gene cluster of SA does not contain a similar gene for citrate synthesis suggesting it must solely rely on the TCA cycle for citric acid production.^51^ Overall, the biosynthesis of the staphyloferrin building blocks (*L*-DAP, α-KG, and citric acid) is a stunning example of evolutionary pressure redirecting biosynthetic pathways.

**Figure 11:**
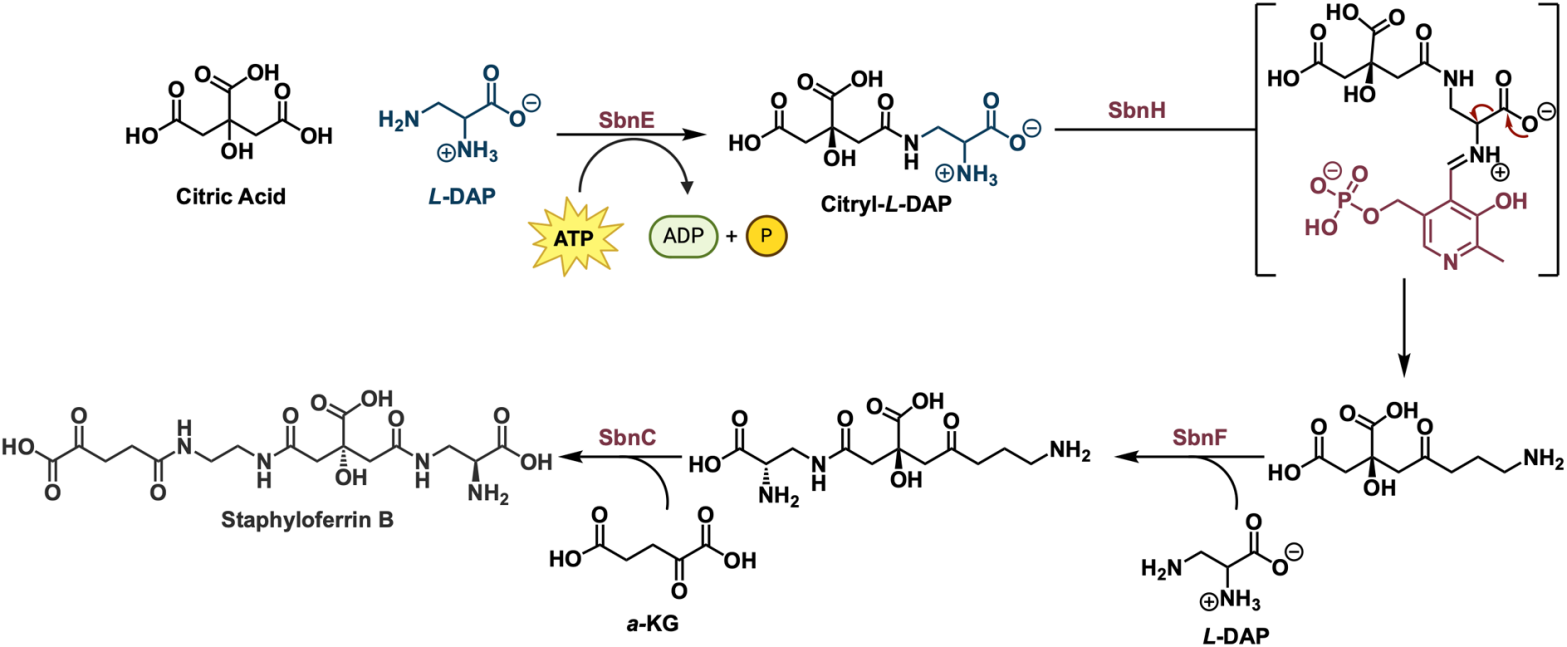
Final biosynthetic steps in the biosynthesis of staphyloferrin B highlighting the PLP-dependent decarboxylation of citryl-*L*-DAP by SbnH.

The biosynthesis of staphyloferrin itself relies on four enzymes SbnE, SbnH, SbnF, and SbnC. SbnE catalyzes an ATP-dependent condensation of *L*-DAP with citric acid to form a citryl-*L*-DAP complex (Figure 11).^57^ This complex then undergoes a PLP-dependent decarboxylation to form the primary amine catalyzed by SbnH.^58^ Following the decarboxylation, the resulting primary amine is then coupled with another molecule of *L-*DAP by the enzyme SbnF.^57^ Finally, SbnC catalyzes the final coupling with α-KG to produce staphyloferrin.

**Figure 12:**
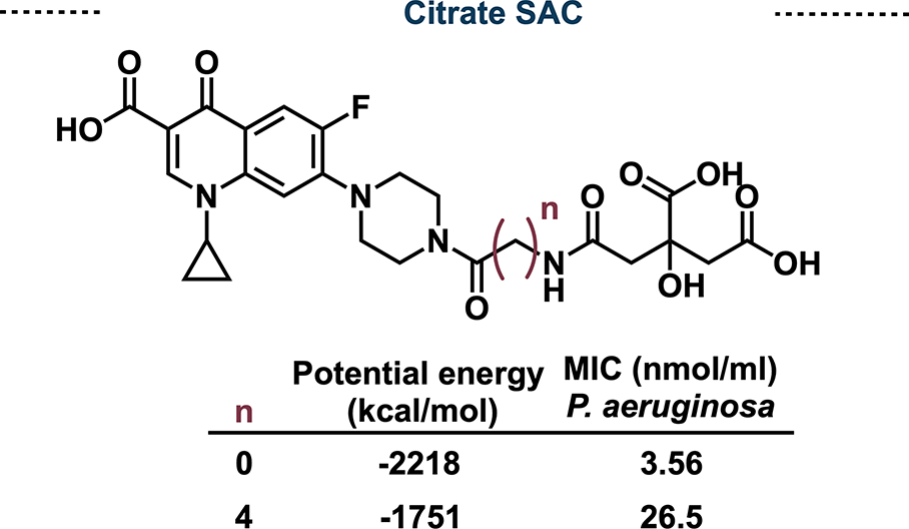
Citrate-ciprofloxacin SACs activity and calculated binding energy.

Similar to other siderophores, the development of staphyloferrin A-citrate antibiotic conjugates has been investigated. Anne-Kathrin Duhme-Klair and Anne Routledge developed citrate-ciprofloxacin conjugates which retained activity but were unable show activity against ciprofloxacin-resistant strains, suggesting that these SACs can reach the target enzyme but are unable to overcome specific resistance mechanisms.^59^ Further studies by the Duhme-Klair Lab showed that the linker length between citrate and ciprofloxacin plays a vital role in antimicrobial activity.^60^ Unlike the cleavable SACs discussed previously, the whole SACs must fit in the active site of the target protein and thus can have drastic effects on binding capabilities and potency. Routledge and Duhme-Klair found that a longer linker length between ciprofloxacin and the citrate motif resulted in lower binding potency, which was validated computationally (Figure 12).^60^ These findings highlight the difficulty in designing SACs that do not alter the native binding. In addition to citric acid conjugates, Duhme-Klair and Routledge probed staphyloferrin A-fluoroquinolone conjugates.^61^ Again, no activity was observed against fluoroquinolone- resistant strains. Moreover, slight changes in the staphyloferrin A structure resulted in complete loss of activity.^61^ Recent work by Nolan (Walsh Lab postdoctoral scholar, 2006–2009) synthesized staphyloferrin B along with the alcohol epimer and a cyclized imide derivative. Intriguingly, they found that the epimer had diminished siderophore activity compared to the natural product. The imide analog was also found to be unstable.^62^

## Catecholate and Phenolate Siderophores: Enterobactin

**Figure 13:**
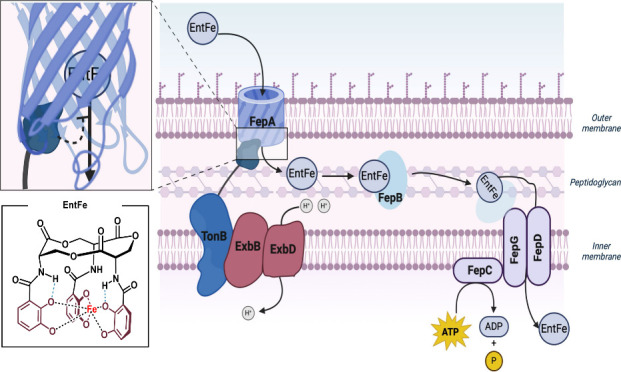
Cellular uptake of the enterobactin-iron complex highlighting the TonB plug within FepA.

Due to their wide occurrence in nature and recent use in SACs, catecholate siderophores are one of the most well studied classes.^63^ Enterobactin (also called enterochelin) is a catecholate siderophore first isolated in 1970 from *Escherichia coli* and S*almonella typhimurium*.^64^ To date, enterobactin (Ent) is the strongest chelating siderophore known with a K_f_ of 10^51^ M^–1^.^65^ The high affinity of Ent can be justified through the hexadentate tris-catecholate chelation with the metal center. In the early 1990s, Kenneth Raymond set out to understand how the chemical structure of Ent allowed for its unique metal binding capabilities. Using X-ray crystallography, Raymond and co-workers crystallized the vanadium(IV)- ent complex and found that the amide backbone linkage allowed for a crucial hydrogen bond between the catechol and amide proton. The additional hydrogen bond prepositioned the three catechol motifs which locks them into place for chelation with the metal center (Figure 13).^66,67^

The uptake and regulation of the Ent-Fe complex has been widely studied. Once iron binds to Ent, the Ent-Fe complex can be transported through the outer membrane of gram-negative bacteria via a 22-stranded barrel protein, FepA.^68^ Interestingly, the intake of Ent-Fe via FepA is regulated by a “plug domain” of the inner membrane protein, Ton B.^69^ TonB protrudes across the periplasm and into the barrel of FepA blocking the intake of Ent-Fe.^70^ TonB is coupled with ExbB and ExbD which utilizes the proton gradient between the periplasm and cytoplasm to displace the TonB plug and allows for Ent-Fe to enter the periplasm.^71^ Initially, it was proposed that the TonB plug was completely removed from the FepA barrel when it allowed Ent-Fe to enter. Later studies showed that the plug domain moved to the side of the FepA barrel allowing Ent-Fe to pass through.^69^ Once the complex is in the cytoplasm it binds with FepB which then directs it to the FepC-FepG-FepD complex, which actively transports Ent-Fe across the inner membrane.^13^ After entry, a final enzyme, Fes, hydrolyzes the Ent-Fe complex to give the unbound siderophore and free iron.^72,73^ Although the intake of each siderophore differs, the general process shown for Ent is found in many other bacteria.^74^

**Figure 14:**
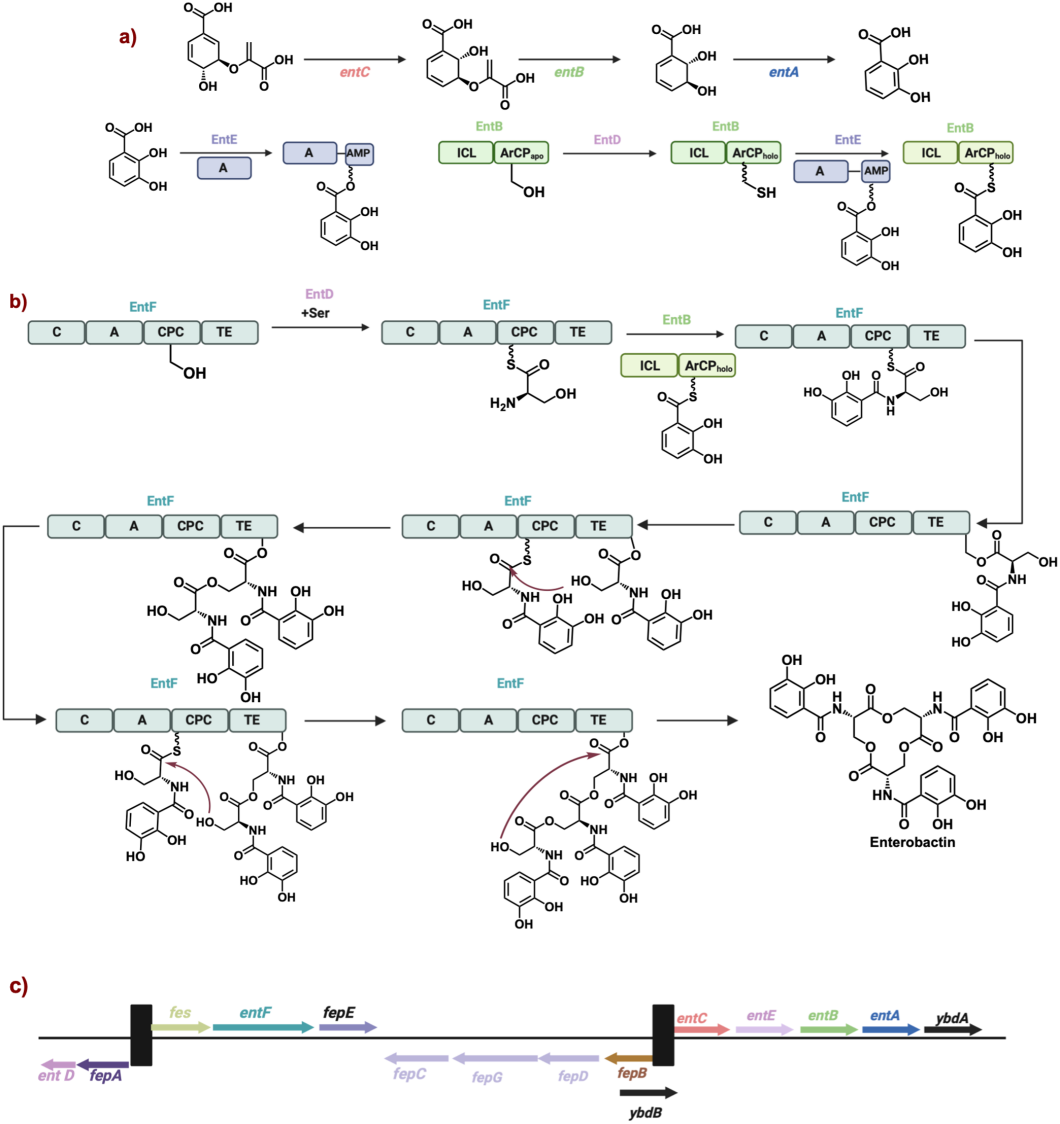
a) Three step biosynthesis of DHB and loading DHB onto EntB. b) The enzymatic assembly line for the synthesis of enterobactin. The biosynthetic gene cluster encoding for enterobactin. (Abbreviations: peptide carrier protein (PCP), adenylation (A). condensation (C), epimerization, and thioesterase (TE). C) The gene clusters encoding for Ent.

The biosynthesis of Ent is well studied and exemplifies the four main steps in of a nonribosomal peptide natural product. Ent possesses three serine residues coupled with three 2,3-dihydroxybenzoate (DHB) motifs. The biosynthesis of DHB relies on three enzymes: EntC, EntB, and EntA (Figure 14a). The first enzyme in this pathway, EntC, is responsible for the reversible conversion of chorismate to isochorismate, analogous to PchA.^75^ Then, a lysis domain on EntB hydrolyzes isochorismate to dihydro-2,3-dihydroxybenzoate and expels pyruvate.^76^ Finally, EntA can perform a rearomatization to furnish DHB. DHB undergoes ligation to form DHB-AMP. Work conducted in the Walsh lab found that EntB not only contained a lysis domain for the synthesis of DHB but also an aryl carrier protein (ArCP) domain to carry DHB.^77^ Initiation begins with the ArCP domain on EntB is phosphopantetheinylated via EntD to give the active EntB_Holo_ domain (Figure 14b). The enterobactin biosynthesis was the seminal example of a phosphopantetheinyl transferase (PPTase) .^78^ This active domain then reacts with the AMP-DHB via EntE to form the DHB adduct. In a separate initiation event, EntF is phosphopantetheinylated by EntD and l-serine is loaded onto the EntF CPC domain.^79^ The EntB-DHB adduct then reacts with the amine of the added serine to produce the coupled Ser-DHB complex on the CPC domain on EntF. The coupled complex is then passed on to the thioesterase (TE) domain. Unlike the biosynthesis of pyochelin, this TE domain is stable to hydrolysis and acts as a holding position for the growing peptide allowing for the CPC domain to make another Ser-DHB complex. The Ser-DHB on the TE domain then engages with newly synthesized Ser-DHB on the CPC domain to form a dimer on the TE domain of EntF.^13^ Interestingly, at this point, there is no self-cleavage to form the cyclic dimer natural product, however this dimer can be detected by tandem mass spectroscopy analysis.^80^ This process repeats itself to finally furnish a trimer on the TE domain which is then terminated via an intramolecular cyclization to produce Ent.

The genes encoding for the biosynthesis ((*entC*, *entB*, *entA*, *entE*, *entD*, and *entF*), cellular intake (*FepA*, *FepB*, *FepC*, *FepD*, *FepG)* and degradation (*fes*) are all clustered together (Figure 14c).^6,82^ Initial genomics data suggested an additional gene, *entG*, however, entG was later found encoded for the lysis domain in EntB.^83^ Taken together, the biosynthesis of enterobactin is a beautiful example of the evolutionary pressure to cluster genes together.

“*My research has always been about what chemical transformation happen in living systems...”*

–Christopher Walsh^81^

**Figure 15:**
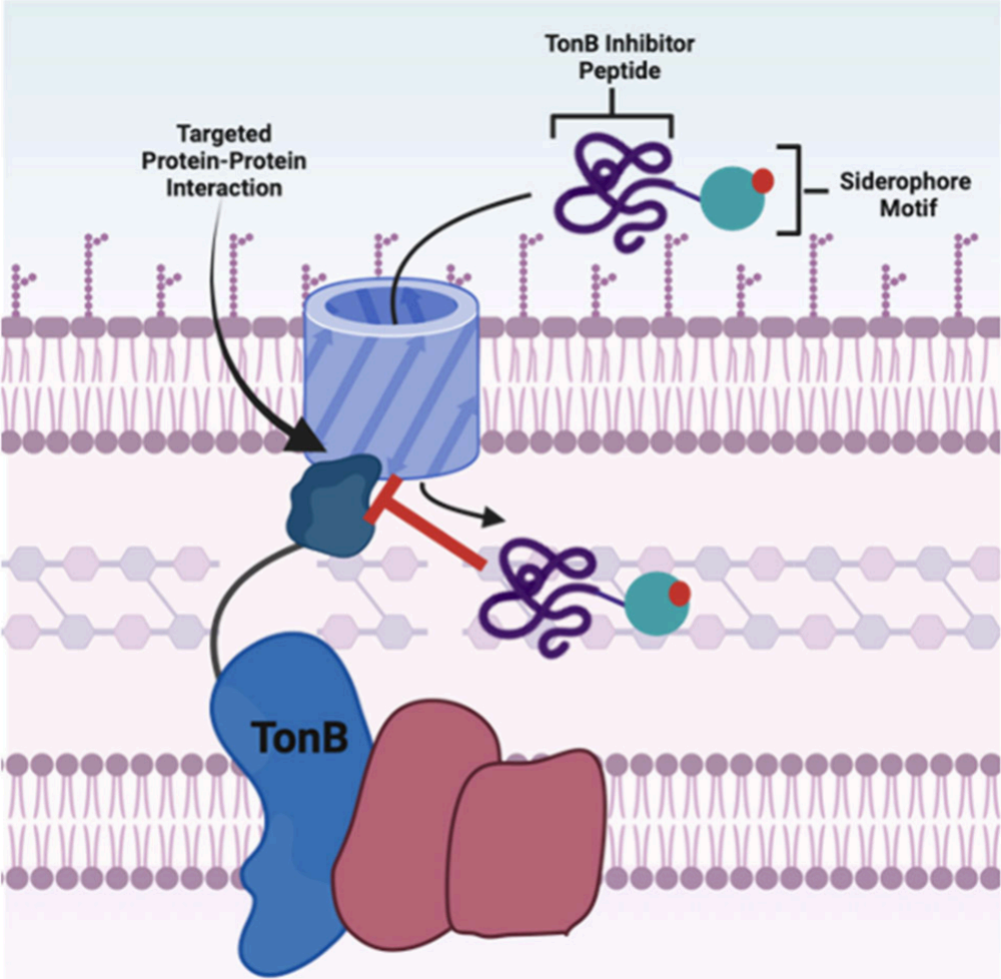
Enterobactin and synthetic Enterobactin mimics.

The fundamental research of enterobactin has led scientists to identify novel antibacterial targets. Specifically, TonB, the regulator protein for outer membrane transport, looked like a promising antibacterial target. By inhibiting TonB, siderophore intake would be inhibited resulting in low levels of iron and eventually downstream cell death. Work by Tuckman Osborne in 1992 showed that a small pentapeptide (ETVIV) that mimics the TonB binding site was able to inhibit TonB activity, albeit at high concentration.^84^ Further work by Braun showed that the *in vivo* synthesis of the entire TonB “plug domain” inhibited siderophore transport, again suggesting that TonB might be a viable antibacterial target (Figure 15).^85^ In 2023, Mark Brönstrup designed a series of TonB peptide inhibitors to inhibit the protein–protein interaction between TonB and outer-membrane transport proteins. However, the peptides alone were too large for passive intake across the outer membrane.^86^ As such, Mark Brönstrup coupled these large peptides to enterobactin mimics to produce novel siderophore-peptide conjugates. These large conjugates, upward of 4 kDa, are transported across the outer-membrane before inhibiting the TonB protein–protein interaction. This strategically designed conjugate targets its own uptake mechanism (Figure 15). The best siderophore-peptide conjugate was found to suppress the growth of *P. aeruginosa* that were unable to produce their own siderophores at concentrations as low as 0.1 μM. This work showed that siderophore conjugates can facilitate the intake of exceptionally large molecules and confirmed that TonB can be an antibiotic target. Overall, this advancement in antibiotic development would not have been possible without the foundational research regarding siderophore uptake, gene regulation, and siderophore biosynthesis.

**Figure 16:**
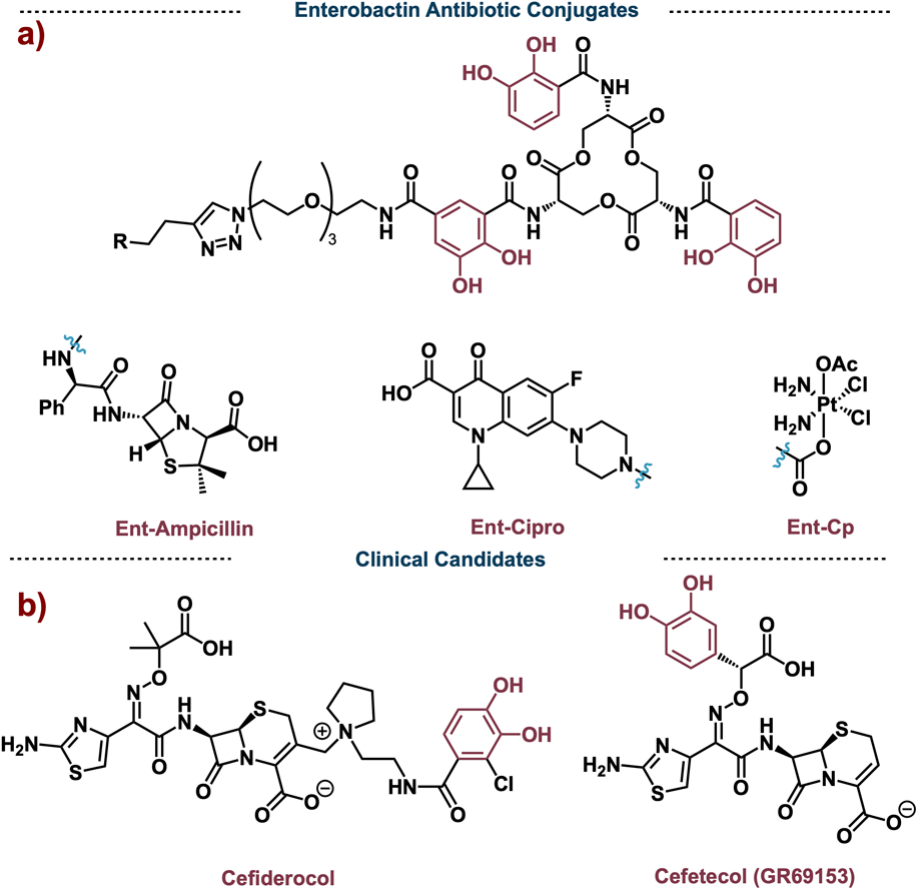
a) Enterobactin-antibiotic conjugates developed in the Nolan Lab. b) Clinical siderophore based candidates.

In addition to these siderophore-peptide conjugates, multiple small molecule Ent antibiotic conjugates have been studied.^87^ Over the years, Nolan developed a wide range of Ent antibiotic conjugates (EAC). In 2012, their lab synthesized and explored Ent-Ciprofloxacin and Ent-Vancomycin conjugates in both *E. coli* and *P. aeruginosa* showing that large EACs can be up taken into the bacterial cytoplasm. Moreover, the cellular intake of EACs differed between *E. coli* and *P. aeruginosa* with *P. aeruginosa* having greater promiscuity for EACs.^88^ Nolan continued to explore EACs and developed ampicillin- and amoxicillin-Ent conjugates. These novel EACs showed up to a 1000-fold decrease in MIC in *E. coli* and killed cells faster than ampicillin or amoxicillin alone.^89^ Ent-ampicillin was also found to exhibit low cytotoxicity to human intestinal cells. Mechanistically, it was found that Ent-ampicillin, like regular enterobactin, is also transported across the outer membrane by FepA.^89^ Nolan continued to be a pioneer in this field, synthesizing nonnative Ent analogs. These synthetically simplified analogs retain activity and still show uptake through the FepA sequence.^90^ Furthermore, these EACs were found to have increased activity against a library of ESKAPE pathogens, bacteria typically associated with causing drug-resistant infections.^90^ Recently, the Nolan lab conjugated enterobactin to cisplatin, a potent anticancer reagent. This novel Ent-Cp conjugate was able to increase platinum (Pt) concentration in *E. coli* 10-fold compared to *E. coli* treated with only cisplatin.^91^ Ent-Cp showed limited Pt uptake in human kidney cells compared to treatment with only cisplatin. This showed that SACs in some sense can direct therapeutics to bacterial cells (Figure 16a).

“*I think the great ‘Aha!’ moments are when you take things from very different fields, or desperate observations, and put them together in a way no one else has before.”*

–Christopher Walsh^81^

Rather than introducing a large complex siderophore to an antibiotic, many research groups have added siderophore-like motifs to an already existing antibiotic in hopes of leveraging the same trojan horse strategy discussed previously. A seminal example of this was in 1990 with the molecule Cefetecol (GR69153). GR69153, which possesses a catechol motif that can bind iron, has potent activity against multiple gram-negative pathogens (MIC < 0.25 μg/mL).^92^ Just like enterobactin, GR69153 hijacks the TonB regulated transport system to pass the outer membrane of ram-negative bacteria.^93^ Although GR69153 never made it to the market, its unique trojan horse mechanism and potent activity launched other pharmaceutical companies including Pfizer,^94^ GSK,^95^ Takeda Pharmaceuticals and Synphar,^96^ Basilea Pharmaceuticals,^97^ and Shionogi Pharmaceuticals^98^ into developing trojan horse antibiotics. Of the many compounds developed, Cefiderocol developed by Shionogi Pharmaceuticals is the first and only cephalosporin siderophore approved for use in the United States and European Union (Figure 16b).^99^ Again, Cefiderocol uses the native machinery of the bacterium to pass through the cellular membrane and deliver the drug. In 2021, Cefiderocol made the WHO list of essential medicines.^100^ Even though Cefiderocol has been used for less than five years, resistant strains have already been reported.^101^ This rapid resistance emphasizes the importance for the continued development and understanding of antibiotics. For a complete review on Cefiderocol and its development, see Syed 2021.^102^

## Beyond Traditional Application

**Figure 17:**
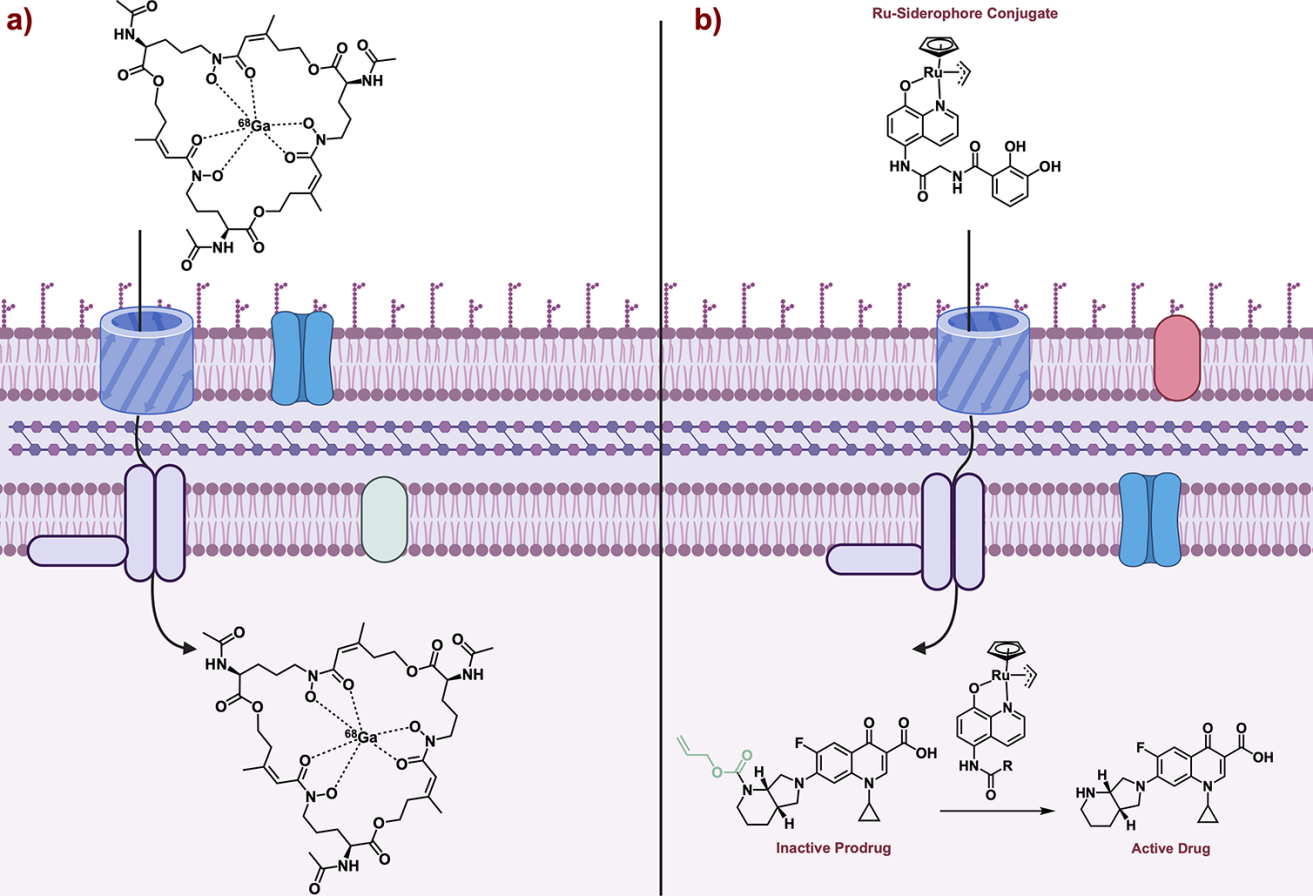
a) Siderophore-gallium complex as an imaging agent. b) Siderophore-ruthenium complex for selective prodrug release.

The translational application of siderophores extends past the development of novel therapeutics for human health. Siderophores have also been applied to other fields including positron emission tomography (PET) scan imaging, chemo-sensors,^103^ artificial metalloenzymes,^104^ and prodrug release mechanisms.^105^ The use of siderophores for PET imaging has recently become an emerging area of research in chemical biology. PET imaging utilizes a radioactive substance, termed a tracer, that emits positrons and as a result allows for imaging within a living system. The use of siderophores in imaging dates back to the late 1970s where Deferoxamine was shown to enhance contrast for gallium-67 imaging by removing gallium in the bloodstream.^106^ Catalyzed by the foundational research regarding siderophores, Decristoforo and workers, hypothesized that replacing iron with radioactive gallium-68 would result in an increase accumulation within bacterial and fungal infections (Figure 17a).^107^ The emission of ^68^Ga would then allow for imaging of the infection *in vivo.* The authors coupled desferri-triacetylfusarinine C, a hydroxamate siderophore, with ^68^Ga and imaged mice with *Aspergillus fumigatus* lung infections.^107^ The siderophore-^68^Ga complex has high biological stability and is selectively taken up by *A. fumigatus* within minutes.^107^ Furthermore, the PET scan was able to identify different levels of infection severity. Taken together, the use of siderophores in imaging has vast clinical potential. More recently, work from Eszter Boros utilized enterobactin-^45^Titanium ^(IV)^ for targeted cancer imaging.^108^ This is only one of many applications of siderophore-assisted imaging. For a complete review of the use of siderophores for molecular imaging applications see Decristoforo, 2017.^109^

The basic science discoveries in the lab of Anne-Kathrin Duhme-Klair led them to explore a siderophore-based prodrug activation strategy.^105^ Traditionally, a prodrug is an inactive compound that is metabolized within a system to produce the active compound. Duhme-Klair hypothesized that they could utilize a catalyst-siderophore conjugate to selectively cleave a prodrug.^105^ To test this hypothesis, they modified moxifloxacin with an allyl carbonate, making it a prodrug (inactive). The allyl carbonate was strategically selected as the “deactivating” group as this moiety has been shown to be cleaved under biological conditions with the treatment of ruthenium.^110^ With this in mind, Duhme-Klair conjugated a ruthenium complex to different siderophores including enterobactin and staphyloferrin mimics. Duhme-Klair showed that the ruthenium-siderophore conjugate could selectively enter bacterial cells along with the moxifloxacin-prodrug. Once both were in the cell, the ruthenium-siderophore conjugate could catalyze the allyl deprotection and release the active compound (Figure 17b).^105^ This prodrug approach was still effective in reducing the growth of bacteria but was unable to outperform moxifloxacin by itself.^105^ This decreased activity was likely due to the catalyst instability and potentially poor cellular uptake.^105^ Nevertheless, this example sets the stage for further translational research into siderophore promoted prodrug activation.^81^

## Conclusion

Siderophore research over the last 50 years, from initial discoveries encompassing biosynthetic machinery, and enzymatic logic to translational applications has been enabled by foundational discoveries. There are still several challenges that lie ahead in the translational research of siderophores. For example, SACs, by definition, require a chemical modification to the antibiotic to introduce the siderophore. This change in structure often diminishes or eliminates all antimicrobial activity rendering the SACs inactive. As highlighted above, cleavable linkers between the antibiotic and siderophore have been explored but still suffer from poor cleavage and efflux. Placing the siderophore motifs directly on small molecules, as in Cefiderocol, have proven to be a possible solution, however, these siderophore motifs are often prone to metabolic degradation. The challenge of balancing siderophore-mediated uptake, antimicrobial activity, and metabolic stability is still a hurdle the community continues to address. There is also a need for more fundamental siderophore research to propel the translational research even further. For instance, the exact structural relationship between the TonB plug domain and TonB inner membrane protein is still unknown. As such, developing rationally designed small molecule inhibitors is extremely difficult. Uncovering the structural relationship between these two domains (basic science) would surely lead to the development of novel inhibitors with potential therapeutic effects (translational research). Overall, siderophore research is a defining example of how basic science catalyzes translational discoveries with substantial impact on human lives. The success of translational siderophore research can be heavily attributed to Walsh’s outlook on science. This approach has been embodied by his mentees who continue to use fundamental research to propel translational advancements in human health.

“*I have been very interested in and committed to translation of basic scientific findings into new therapeutic agents...”*

–Christopher Walsh^81^

## Dedication

This review is dedicated to the life of Prof. Christopher T. Walsh, a beloved mentor and advisor to >250 trainees over his 40+ year career. His scientific impact is immeasurable, essentially defining an entire new subfield of chemical and biological research. W.M.W. is forever indebted to the impact Chris had on his career and all his advice and support.
